# Supplementary figures and images for: Study on SARS-CoV-2 infection in middle-aged and elderly population infected with hepatitis virus: a cohort study in a rural area of northeast China
Source: PeerJ. 2025 Feb 21;13:e19021. doi: 10.7717/peerj.19021 (PMC11849502; doi:10.7717/peerj.19021)

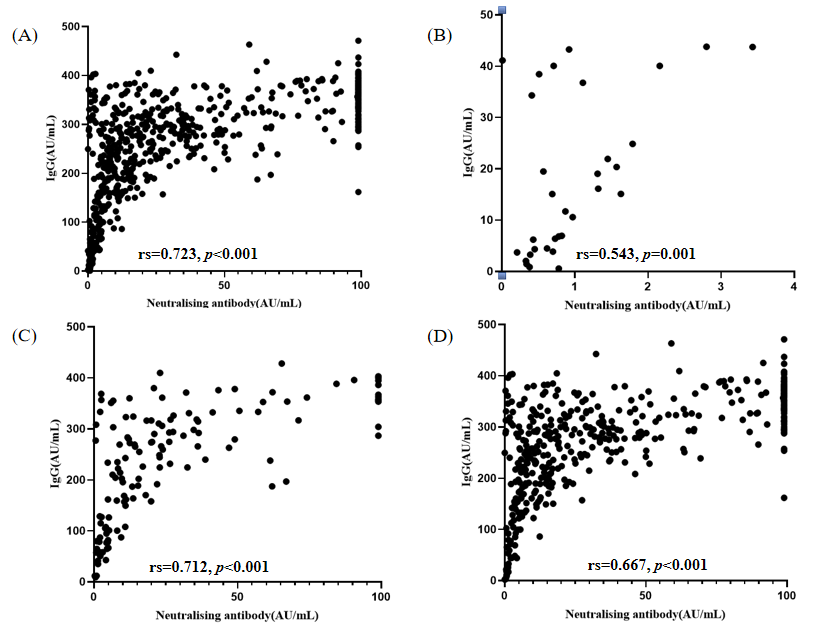

Supplement: Supplemental Information 1 — (A) Total patients; (B) Uninfected; (C) Asymptomatic; (D) Symptomatic. [file peerj-13-19021-s001.png]
