# Supplementary material for: Study on SARS-CoV-2 infection in middle-aged and elderly population infected with hepatitis virus: a cohort study in a rural area of northeast China
Source: PeerJ. 2025 Feb 21;13:e19021. doi: 10.7717/peerj.19021 (PMC11849502; doi:10.7717/peerj.19021)
Supplement: Supplemental Information 2 [file peerj-13-19021-s002.docx]

Supplementary TableS1. Methods of liver function tests and HCV RNA

| Index | Method | Units | Reference range |
| --- | --- | --- | --- |
| ALT | Rate analytical method | U/L | Male: 9.0-50.0  Female: 7.0-40.0 |
| AST | Rate analytical method | U/L | Male:15.0-40.0  Female:13.0-35.0 |
| GGT | Rate analytical method | U/L | Male:10.0-60.0  Female:7.0-45.0 |
| ALP | Rate analytical method | U/L | Male: 45.0-125.0  Female:50.0-135.0 |
| LDH | Rate analytical method | U/L | 120-250 |
| TBIL | Vanadate oxidation method | μmol/L | Male: 0.00-26.00  Female:0.00-21.00 |
| DBIL | Vanadate oxidation method | μmol/L | 0.00-8.00 |
| HCV RNA | RT-qPCR | IU/mL | <5.00E+02 |
