# Supplementary material for: Study on SARS-CoV-2 infection in middle-aged and elderly population infected with hepatitis virus: a cohort study in a rural area of northeast China
Source: PeerJ. 2025 Feb 21;13:e19021. doi: 10.7717/peerj.19021 (PMC11849502; doi:10.7717/peerj.19021)
Supplement: Supplemental Information 3 [file peerj-13-19021-s003.docx]

Supplementary TableS2. Symptoms after infection in HCC high risk patients

| Symptomatic | n=435 |
| --- | --- |
| Fever | 228(52.4) |
| Cough | 180(41.4) |
| Myalgia/Joint pain | 129(29.6) |
| Sore throat | 65(14.9) |
| General malaise | 62(14.3) |
| Fatigue | 60(13.8) |
| Headache | 52(11.8) |
| Congestion/Runny nose | 17(3.9) |
| Dyspnea/Chest distress | 15(3.4) |
| Celialgia/Diarrhea | 8(1.8) |
| Nausea/Vomiting | 6(1.4) |
| Loss of taste | 5(1.1) |
| Loss of smell | 5(1.1) |
| Sleep disturbance | 1(0.2) |
| Other | 7(1.6) |
