# Supplementary material for: Study on SARS-CoV-2 infection in middle-aged and elderly population infected with hepatitis virus: a cohort study in a rural area of northeast China
Source: PeerJ. 2025 Feb 21;13:e19021. doi: 10.7717/peerj.19021 (PMC11849502; doi:10.7717/peerj.19021)
Supplement: Supplemental Information 4 [file peerj-13-19021-s004.docx]

**Supplementary TableS3. Comparison of liver function at different time points and different infection states**

| Variable | pre | | *p* | post | | *p* | OR(95%CI)  (post/pre) | *p* | OR(95%CI)  (infected/uninfected) | *p* |
| --- | --- | --- | --- | --- | --- | --- | --- | --- | --- | --- |
|  | uninfected | infected |  | uninfected | infected |  |  |  |  |  |
| ALT(male>50U/L;  female>40U/L) | 2(6.5) | 46(8.7) | 0.762 | 1(3.1) | 33(5.5) | 0.719 | 0.60(0.39-0.92) | 0.018 | 1.33(0.29-6.08) | 0.715 |
| AST(male>40U/L;  female>35U/L) | 3(9.7) | 77(14.6) | 0.602 | 3(9.4) | 44(7.8) | 0.732 | 0.51(0.37-0.71) | <0.001 | 1.25(0.41-3.82) | 0.698 |
| GGT(male>60U/L;  female>45U/L) | 7(22.6) | 78(14.8) | 0.298 | 6(18.8) | 96(16.9) | 0.809 | 1.16(0.95-1.43) | 0.152 | 0.62(0.26-1.45) | 0.266 |
| ALP(male>125U/L;  female>135U/L) | 4(12.9) | 29(5.5) | 0.102 | 2(6.3) | 37(6.5) | 1.000 | 1.05(0.75-1.46) | 0.784 | 0.92(0.28-3.03) | 0.886 |
| LDH(>250 U/L) | 9(29.0) | 115(21.8) | 0.373 | 4(12.5) | 48(8.5) | 0.512 | 0.32(0.24-0.44) | <0.001 | 0.96(0.46-2.01) | 0.914 |
| TBIL(male>26μmol/L  female>21μmol/L) | 1(3.1) | 11(2.1) | 0.651 | 2(6.3) | 14(2.5) | 0.208 | 1.39(0.69-2.82) | 0.354 | 0.51(0.12-2.27) | 0.377 |
| DBIL(>8μmol/L) | 2(6.5) | 26(4.9) | 0.663 | 2(6.3) | 38(6.7) | 1.000 | 1.43(0.96-2.13) | 0.080 | 0.83(0.23-2.93) | 0.769 |
