# Supplementary material for: Study on SARS-CoV-2 infection in middle-aged and elderly population infected with hepatitis virus: a cohort study in a rural area of northeast China
Source: PeerJ. 2025 Feb 21;13:e19021. doi: 10.7717/peerj.19021 (PMC11849502; doi:10.7717/peerj.19021)
Supplement: Supplemental Information 5 [file peerj-13-19021-s005.docx]

**Supplementary TableS4.** Comparison of aMAP score at different time points and

different infection states

|  | pre | | *p* | post | | *p* | B  (post-pre) | Std.err | *p* | B  (infected-uninfected) | Std.err | *p* |
| --- | --- | --- | --- | --- | --- | --- | --- | --- | --- | --- | --- | --- |
|  | uninfected | infected |  | uninfected | infected |  |  |  |  |  |  |  |
| aMAP | 55(49-59) | 53(49-57) | 0.413 | 55(51-61) | 54(50-58) | 0.212 | 0.893 | 0.107 | <0.001 | -0.423 | 0.672 | 0.529 |
