# Supplementary material for: Study on SARS-CoV-2 infection in middle-aged and elderly population infected with hepatitis virus: a cohort study in a rural area of northeast China
Source: PeerJ. 2025 Feb 21;13:e19021. doi: 10.7717/peerj.19021 (PMC11849502; doi:10.7717/peerj.19021)
Supplement: Supplemental Information 6 [file peerj-13-19021-s006.docx]

**Supplementary TableS5. Comparison of aMAP score of cirrhosis patients at different time points and different infection states**

|  | pre | | *p* | post | | *p* | B  (post-pre) | Std.err | *p* | B  (infected-uninfected) | Std.err | *p* |
| --- | --- | --- | --- | --- | --- | --- | --- | --- | --- | --- | --- | --- |
|  | uninfected | infected |  | uninfected | infected |  |  |  |  |  |  |  |
| aMAP | 58(55-67) | 56(53-62) | 0.237 | 61(57-67) | 58(54-62) | 0.170 | 0.949 | 0.305 | 0.002 | 4.040 | 2.458 | 0.100 |
