# Supplementary material for: Study on SARS-CoV-2 infection in middle-aged and elderly population infected with hepatitis virus: a cohort study in a rural area of northeast China
Source: PeerJ. 2025 Feb 21;13:e19021. doi: 10.7717/peerj.19021 (PMC11849502; doi:10.7717/peerj.19021)
Supplement: Supplemental Information 7 [file peerj-13-19021-s007.docx]

**Supplementary TableS6.** Long-covid 19 symptoms in HCC high risk patients

| Long-covid symptoms | 148(26.1) |
| --- | --- |
| Fatigue | 67(11.8) |
| Cough | 25(4.4) |
| Myalgia/ Joint pain | 19(3.4) |
| Hypomnesia | 12(2.1) |
| Dyspnea/ asthma | 12(2.1) |
| Chest pain | 8(1.4) |
| Sore throat/ dysphagia | 7(1.2) |
| Loss of appetite | 7(1.2) |
| Tachycardia | 7(1.2) |
| Headache/ dizziness | 5(0.9) |
| Sleep disturbance | 4(0.7) |
| Loss of taste | 4(0.7) |
| Loss of smell | 2(0.4) |
| Nausea/Vomiting | 1(0.2) |
| Other | 17(3.0) |
