# Supplementary material for: Study on SARS-CoV-2 infection in middle-aged and elderly population infected with hepatitis virus: a cohort study in a rural area of northeast China
Source: PeerJ. 2025 Feb 21;13:e19021. doi: 10.7717/peerj.19021 (PMC11849502; doi:10.7717/peerj.19021)
Supplement: Supplemental Information 8 [file peerj-13-19021-s008.docx]

**Supplementary TableS7.** Comparison of neutralizing antibody and IgG in different groups.

|  |  | neutralizing antibody(AU/mL) | IgG(AU/mL) |
| --- | --- | --- | --- |
| long-COVID | Yes | 25.40(9.85-85.59) | 287.12(191.71-358.71) |
|  | No | 20.30(7.44-59.92) | 288.14(211.04-337.42) |
| *p* |  | 0.119 | 0.797 |
| reinfection | Yes | 18.03(7.57-49.02) | 268.62(179.05-328.76) |
|  | No | 22.93(8.02-62.04) | 286.49(210.02-339.68) |
| *p* |  | 0.477 | 0.148 |
