# Supplementary material for: Study on SARS-CoV-2 infection in middle-aged and elderly population infected with hepatitis virus: a cohort study in a rural area of northeast China
Source: PeerJ. 2025 Feb 21;13:e19021. doi: 10.7717/peerj.19021 (PMC11849502; doi:10.7717/peerj.19021)
Supplement: Supplemental Information 9 [file peerj-13-19021-s009.docx]

**Supplementary TableS8 . Univariate and multivariate logistic regression analyses of factors to long COVID-19**

|  | Univariate Logistic-regression | | Multivariate Logistic-regression | |
| --- | --- | --- | --- | --- |
|  | OR(95%CI) | *P* | OR(95%CI) | *P* |
| Age(years) | 1.01(0.98-1.03) | 0.661 | 1.01(0.98-1.04) | 0.498 |
| Gender(F/M) | 1.53(1.02-2.28) | 0.040 | 1.49(0.99-2.25) | 0.057 |
| NO. of Vaccination received |  |  |  |  |
| ≤1 | ref |  | ref |  |
| 2 | 0.25(0.37-1.72) | 0.160 | 0.22 (0.03-1.56) | 0.130 |
| 3 | 0.34(0.56-2.07) | 0.242 | 0.31(0.05-1.93) | 0.212 |
| Hepatitis virus infection |  |  |  |  |
| Only HCV infected | ref |  | ref |  |
| Only HBV infected | 1.44(0.72-2.87) | 0.300 | 1.46(0.71-3.02) | 0.302 |
| HCV& HBV co-infected | 4.22(1.04-17.17) | 0.044 | 3.94(0.92-16.95) | 0.065 |
| Liver cirrhosis(Yes/No) | 1.51(0.81-2.84) | 0.196 | 1.21(0.62-2.38) | 0.574 |
