# Supplementary material for: Study on SARS-CoV-2 infection in middle-aged and elderly population infected with hepatitis virus: a cohort study in a rural area of northeast China
Source: PeerJ. 2025 Feb 21;13:e19021. doi: 10.7717/peerj.19021 (PMC11849502; doi:10.7717/peerj.19021)
Supplement: Supplemental Information 10 [file peerj-13-19021-s010.docx]

**Supplementary TableS9 . Univariate and multivariate logistic regression analyses of factors to reinfection**

|  | Univariate Logistic-regression | | Multivariate Logistic-regression | |
| --- | --- | --- | --- | --- |
|  | OR(95%CI) | *P* | OR(95%CI) | *P* |
| Age(years) | 0.96(0.93-0.99) | 0.039 | 0.96(0.93-1.01) | 0.059 |
| Gender(F/M) | 1.03(0.61-1.74) | 0.920 | 1.10(0.63-1.93) | 0.728 |
| Vaccination(Yes/No) | 1.72(0.69-4.29) | 0.243 | 1.79(0.71-4.49) | 0.214 |
| Liver cirrhosis(Yes/No) | 0.53(0.20-1.38) | 0.194 | 0.38(0.11-1.30) | 0.122 |
| Chronic underlying conditions(Yes/No) | 1.17(0.68-1.99) | 0.571 | 1.41(0.80-2.49) | 0.237 |
| Neutralizing antibody (AU/mL) | 0.99(0.99-1.01) | 0.440 | 1.00(0.99-1.01) | 0.981 |
| IgG (AU/mL) | 0.99(0.99-1.01) | 0.155 | 0.99(0.99-1.01) | 0.112 |
